# Pregnancy and lactation‐related osteoporosis in a 22‐year‐old‐woman

**DOI:** 10.1002/ccr3.8489

**Published:** 2024-02-12

**Authors:** Ali Ghassa, Yara Hodifa, Qais Falhout

**Affiliations:** ^1^ Faculty of Medicine Damascus University Damascus Syria; ^2^ Department of Rheumatology, Alassad Univeristy Hospital Damascus University Damascus Syria; ^3^ Department of Orthopedic Surgery Zaid Al Sharity Hospital As Suwayda Syria

**Keywords:** lactation, osteoporosis, pregnancy

## Abstract

**Key Clinical Message:**

Any pregnant or lactating woman with severe constant back pain, PLO must be kept in mind due to its effect on the quality of life of the mother and her child.

**Abstract:**

A 22‐year‐old woman, who delivered her first child 5 months ago and is now breastfeeding her baby, presented with mid‐back pain. After investigations, including laboratory tests, X‐rays, and bone density measurements, the diagnosis was PLO. The patient is being treated with calcium, vitamin D, and alendronate besides discontinuation of lactation.

## 1 INTRODUCTION

Osteoporosis is a global public health problem. Although postmenopausal osteoporosis is the most common type, more than 50% of premenopausal women have secondary osteoporosis.^1^ Pregnancy and lactation‐associated osteoporosis (PLO) is an uncommon condition of skeletal fragility that affects women during pregnancy or postpartum period. It presents with back pain and vertebral compression fractures.^2^ The management of PLO is challenging, but early diagnosis, stopping breastfeeding, treatment of anti‐osteoporosis medicine, and regular follow‐ups are necessary for the prevention of fractures and to increase the mother's quality of life.^3^, ^4^ We present a very rare case of a 22‐year‐old woman, who has been diagnosed with PLO 5 months after delivering her child.

## 2 CASE PRESENTATION

A 22‐year‐old woman, gravida 1, para 1, presented to the clinic with a 5‐month mid‐back pain that started 40 days after delivering her child without any history of trauma. The pain radiates to the lower back and the buttocks and is mechanic characteristically. The patient mentioned no back pain or any of the aforementioned symptoms prior to pregnancy, as well as normal pregnancy and delivery conditions. She is a housewife, who did not use to suffer from any difficulties doing the house chores. The patient used to take supplements during prenatal period, besides that no laboratory checkup was done during that phase. The nutritional status before and during pregnancy was normal. She is now breastfeeding her child. Her history and her family history are unremarkable, especially for osteoporosis. The patient does not use tobacco, alcohol, or any chronic medications.

The physical examination showed kyphosis in the middle of the vertebral column, with tenderness when palpating the thoracic and lumbar vertebral processus and perivertebral muscles. There is a painful limitation of the vertebral column movements. The rest of the joints were normal on examination. The neurological examination also showed no abnormalities. The patient weighs 53 kg, and her height is 152 cm (BMI = 22.9 kg/m^2^).

**FIGURE 1:**
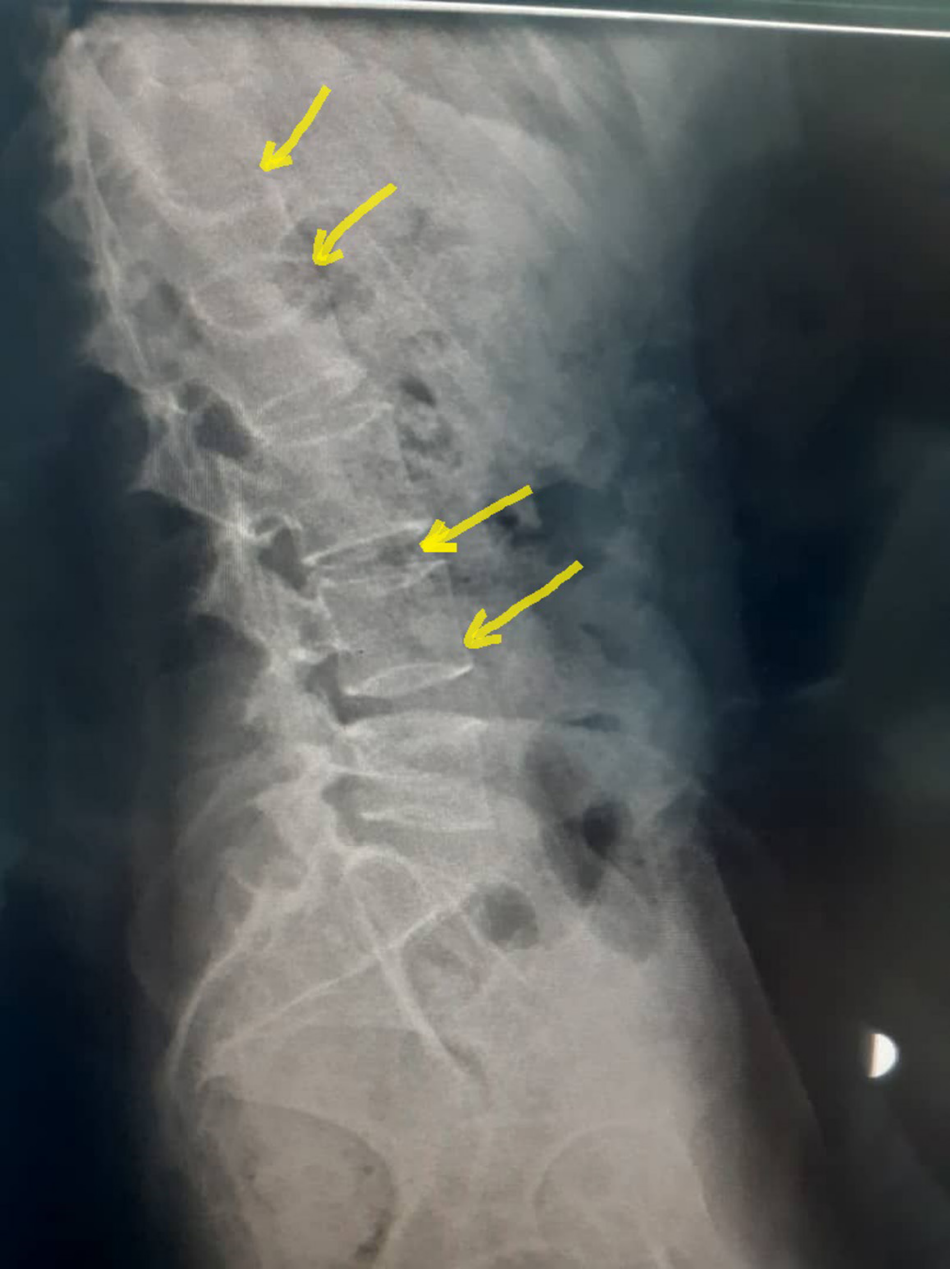
Spine X‐ray showing the wedge vertebrae.

**FIGURE 2:**
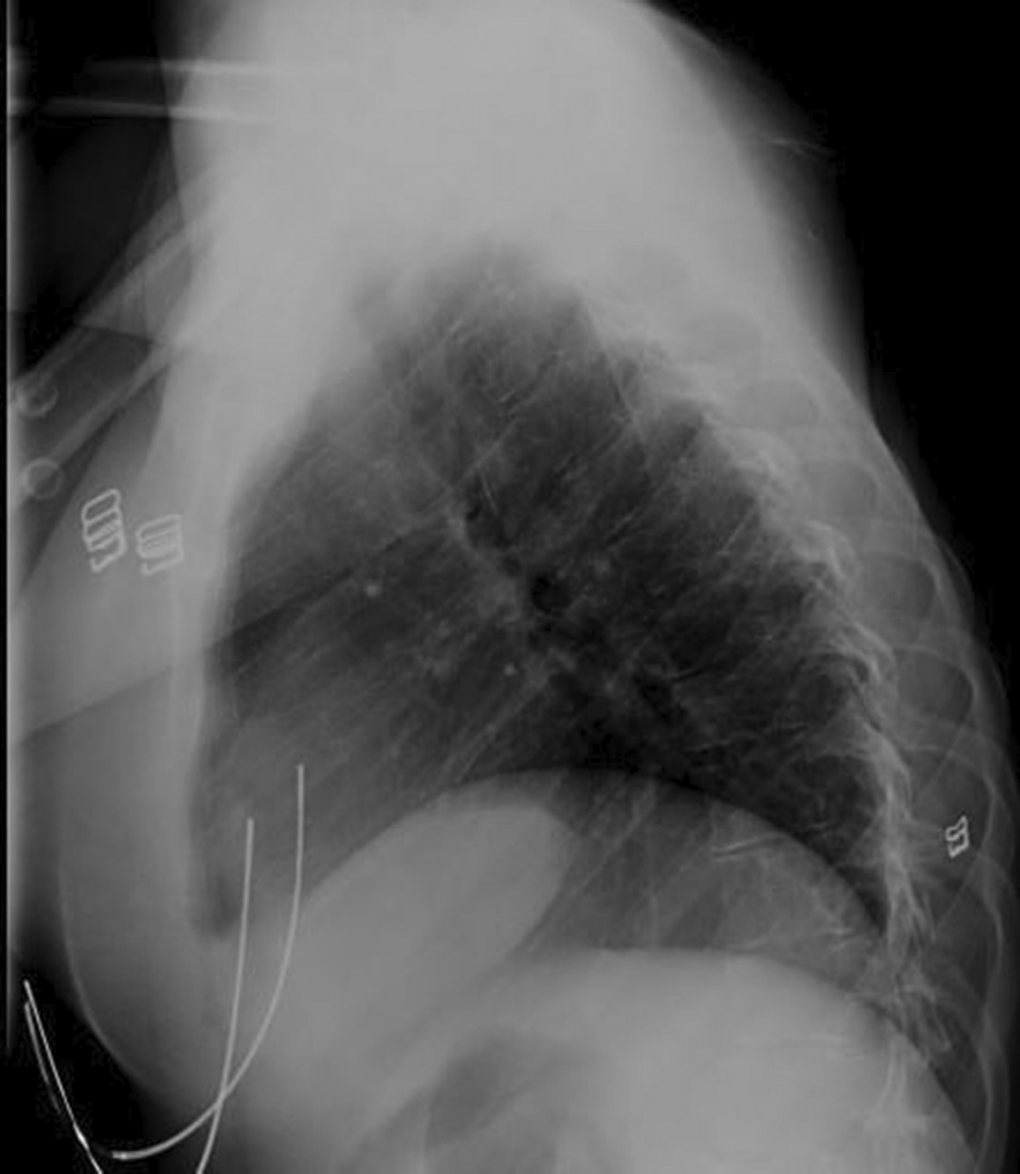
X‐ray showing the kyphosis appearance.

Investigations started with laboratory tests (shown in Table 1) and a spine X‐ray (Figures 1 and 2). Laboratory test values were within the normal range, but the X‐ray showed wedge vertebrae in the middle of the vertebral column. These two combined guided us to do bone density measurements for the vertebral column and the left femoral neck (values shown in Tables 2 and 3), which confirmed the diagnosis of osteoporosis and a high risk for fractures in the spine and osteopenia in the femoral neck with increased risk for fractures. After a workup, the final diagnosis was pregnancy and lactation‐related osteoporosis.

**TABLE 1:** Laboratory tests.

Laboratory test	Test value
Hemoglobin	13 mg/dL
Hematocrit	39.5%
RBCs	4.94 × 10^9^/mm^3^
MCV	80 fL
MCHC	32.9 g/dL
MCH	26.3 pg
WBC	6.58 × 10^3^/mm^3^
Platelets	294,000/mm^3^
ESR	12 mm/h
CRP	0.25 mg/dL
Creatinine	0.71 mg/dL
Calcium	9.4 mg/dL
Phosphorus	4 mg/dL
Total protein	8.1 g/dL
Albumin	6 g/dL
ALT	12 U/L
AST	19 U/L
Alkaline phosphate	116 U/L
25 OH Vitamin D	27.5 ng/mL
Parathyroid hormone	27.7 pg/mL
TSH	0.87 uUl/ml
Magnesium	2.16 mg/dL

**TABLE 2:** DXA of the spine.

Region	Area (cm^2^)	BMC (g)	BMD (g/cm^2^)	*T*‐score	PR (%)	*Z*‐score	AM (%)
L1	11.87	8.76	0.738	−2.3	74	−2.2	76
L2	13.11	9.36	0.714	−2.9	69	−2.7	71
L3	15.31	10.18	0.665	−3.8	61	−3.6	62
L4	13.49	13.49	0.650	−3.7	61	−3.6	62
Total	53.78	37.07	0.689	−3.3	66	−3.1	67

**TABLE 3:** DXA of the femoral neck.

Region	Area (cm^2^)	BMC (g)	BMD (g/cm^2^)	*T*‐score	PR (%)	*Z*‐score	AM (%)
Neck	4.97	3.51	0.706	−1.3	83	−1.3	83
Total	32.53	21.93	0.674	−2.2	72	−2.2	72

The patient is treated with vitamin D 1000 IU daily, calcium 1200 mg per day, and alendronate 70 mg weekly. Besides that, the patient has been told to discontinue breastfeeding. After 5 months of follow‐up, the patient declared symptoms relief including pain relief and better movement.

There are some limitations of the case, such as not doing hormonal tests due to the financial status of the patient.

## 3 DISCUSSION

Pregnancy lactation‐related osteoporosis (PLO) is a very rare type of osteoporosis that occurs in the third term of pregnancy or after delivery.^5^ It happens approximately 4–8 times per million delivery.^5^ It was mentioned for the first time by Nordin Roper in 1955.^6^ There are around 100 documented cases in the world.^7^

The patients usually present with severe low back pain and less likely femoral or pelvic pain.^5^ There are no obvious criteria to diagnose PLO, but a diagnosis can be made with typical signs of osteoporosis, such as spontaneous vertebral fractures and low bone density during or after pregnancy, in addition to ruling out the secondary causes of osteoporosis, including hematological, endocrinal, rheumatologic, renal, and gastroenterological causes besides medications.^5^

The etiology is not clear yet, but it is explained as the following: 3% of maternal calcium deposit passes to the fetus during pregnancy through placenta.^8^ The calcium transportation average increases as the fetus gets older, so the mother's need for calcium increases during pregnancy,^6^ this is the reason why Intestinal absorption of calcium doubles during pregnancy, which is considered the main mechanism, besides the help of bone resorption, yet the loss of bone density remains little [hardcastler]. In lactation, bone resorption is the main mechanism to provide the calcium when breastfeeding, done by the osteoclast's activity through OPG‐RANK‐RANKL and parathormone‐related peptide (PTHrP) which is secreted from the breasts.^6^, ^7^ So, in normal pregnancies, loss of bone density is expected, but it is temporary and reversible, and no complications develop neither at the same time nor in the long term.^9^

Risk factors of PLO include genetic factors, physical activity, and immobilization during pregnancy, low body mass index (BMI), smoking, vitamin D deficiency, alcohol, hypothyroidism, family history, previous traumas, and medications such as corticosteroids, heparin, and anticonvulsant drugs.^7^, ^9^, ^10^, ^11^, ^12^ The most affected area is the thoracolumbar region.^11^

The treatment goal is the prevention of new vertebral fractures, pain relief, and increasing bone matrix density (BMD), and should be planned for each patient on her own depending on age, disease severity, and later pregnancy plans.^6^ The treatment is either conservative or through medications. Conservative management is through lactation discontinuation, calcium, and vitamin D supplements, avoiding heavy weight lifting, lifestyle modification, a balanced diet, and regular exercise.^6^, ^8^

Medications used to treat osteoporosis include bisphosphonate. There are no randomized trials to ensure the efficacy of the bisphosphonate on PLO compared with those, who did not take the drug, but according to small trials, they found that symptoms such as pain and limited movement have resolved faster. In a study on the use of bisphosphonate before and during pregnancy, they concluded that there is no evident risk on the mother or the fetus. However, few case reports revealed some side effects like shortened gestational age, low birth weight, and transient neonatal hypocalcemia.^9^ Denosumab is a human monoclonal antibody that binds receptor activator nuclear factor kappa ligand (RANKL) that works by inhibiting osteoclasts and has good outcomes.^6^, ^11^ Teriparatide is a parathyroid hormone (PTH) analogue used to treat osteoporosis, and there is one advantage, that it does not accumulate in the bone skeleton and is not likely to affect the fetus after it is corrupted.^13^

## 4 CONCLUSION

Since PLO is not common and has no criteria to be diagnosed, any pregnant or lactating woman with severe constant back pain or low back pain, PLO should be suspected. PLO has a strong effect on the quality of life, leads to psychological stress, and might affect caring for her child.

## AUTHOR CONTRIBUTIONS

**Ali Ghassa:** Conceptualization; writing – original draft; writing – review and editing. **Yara Hodifa:** Writing – original draft. **Qais Falhout:** Writing – original draft.

## FUNDING INFORMATION

The authors received no funding regarding the publication of this article.

## CONFLICT OF INTEREST STATEMENT

The authors declare no conflicts of interest.

## CONSENT

Written informed consent was obtained from the patient to publish this report in accordance with the journal's patient consent policy.

## Data Availability

All data generated during this study can be accessed through direct communication with the corresponding author and the agreement of all research team members.
